# Research of Multimodal Medical Image Fusion Based on Parameter-Adaptive Pulse-Coupled Neural Network and Convolutional Sparse Representation

**DOI:** 10.1155/2020/3290136

**Published:** 2020-01-24

**Authors:** Jingming Xia, Yi Lu, Ling Tan

**Affiliations:** ^1^School of Electronics and Information Engineering, Nanjing University of Information Science & Technology, Nanjing 210044, China; ^2^School of Computer and Software, Nanjing University of Information Science & Technology, Nanjing 210044, China

## Abstract

Visual effects of medical image have a great impact on clinical assistant diagnosis. At present, medical image fusion has become a powerful means of clinical application. The traditional medical image fusion methods have the problem of poor fusion results due to the loss of detailed feature information during fusion. To deal with it, this paper proposes a new multimodal medical image fusion method based on the imaging characteristics of medical images. In the proposed method, the non-subsampled shearlet transform (NSST) decomposition is first performed on the source images to obtain high-frequency and low-frequency coefficients. The high-frequency coefficients are fused by a parameter‐adaptive pulse-coupled neural network (PAPCNN) model. The method is based on parameter adaptive and optimized connection strength *β* adopted to promote the performance. The low-frequency coefficients are merged by the convolutional sparse representation (CSR) model. The experimental results show that the proposed method solves the problems of difficult parameter setting and poor detail preservation of sparse representation during image fusion in traditional PCNN algorithms, and it has significant advantages in visual effect and objective indices compared with the existing mainstream fusion algorithms.

## 1. Introduction

The diversity of image capture mechanisms allows different patterns of medical images to reflect different organ and tissue information categories. For example, computed tomography (CT) is very sensitive to blood vessels and bones and thus its imaging is more clearly. Magnetic resonance imaging (MRI) images provide richer soft-tissue information but lack boundary information and blur the bone imaging [1]. Emission computed tomography (ECT), which includes positron emission tomography (PET) and single-photon emission computed tomography (SPECT), captures projected data and reconstructs tomography images with high sensitivity but low resolution. The purpose of pixel-level medical image fusion technology is to obtain more useful and accurate medical information for the same target by combining the complementary information in multimodal medical images through composite image.

In recent years, medical image fusion algorithms have been greatly developed. However, most medical image fusion methods adopt the framework of multiscale transform (MST) to achieve better results. The image transformation method and the fusion strategy of high-frequency coefficients and low-frequency coefficients are the two key issues of MST-based fusion methods. A large number of studies have shown that the performance of MST-based fusion methods can be significantly improved by selecting appropriate image transform methods and designing effective fusion strategies. Singh et al. [2] proposed to add the pulse‐coupled neural network (PCNN) to the fusion rule under the NSST framework to effectively extract the gradient features and preserve the edge and detail information of the source images, but many parameter settings in PCNN are also a major challenge. Liu et al. [3] raised a convolutional sparse representation algorithm, which properly solved the two problems of sparse representation arising in image fusion, i.e., limited ability to preserve details and high sensitivity to registration errors [4, 5] and accomplished the image fusion by implementing a sparse representation of the entire image. Chen et al. [6] proposed an image segmentation method based on a simplified PCNN model (SPCNN). This model can automatically set the size of PCNN-free parameters to achieve higher segmentation accuracy. Ming et al. [7] improved the SPCNN model and obtained an improved parameter‐adaptive PCNN model (PAPCNN) and applied it to image fusion. Experiments showed that the PAPCNN model has a faster convergence rate as well as a preferable effect when applied to the image fusion experiment.

Aiming at the problems existing in the current PCNN and NSST methods, an NSST-PAPCNN-CSR algorithm combining NSST, CSR, and PAPCNN models was proposed. The innovations of this paper are outlined as follows:

1. We adopt the parameter‐adaptive PCNN (PAPCNN) to fuse high-frequency coefficients with all the PCNN parameters adaptively calculated based on the input bands, which can overcome the difficulty of setting free parameters in the conventional PCNN models. Besides, we propose an improved implicit parameter *β* of PAPCNN, and the synchronous ignition characteristics in the PAPCNN model were coordinated to achieve a better fusion effect.
2. We introduce the convolutional sparse representation (CSR) model into the fusion of low-frequency coefficients. The CSR model overcomes the two key issues of sparse representation arising in image fusion, i.e., limited ability to preserve details and high sensitivity to registration errors. In addition, the CSR is expected to solve the sparseness problem of the low-frequency coefficients in the NSST domain.

The rest of this paper is organized as follows. In Section 2, materials and methods used in the paper are briefly introduced.  Section 3 gives the experiments and analysis. Finally, this paper is concluded in Section 4.

## 2. Materials and Methods

### 2.1. Related Materials

#### 2.1.1. Non-Subsampled Shearlet Transform (NSST)

**Figure 1:**
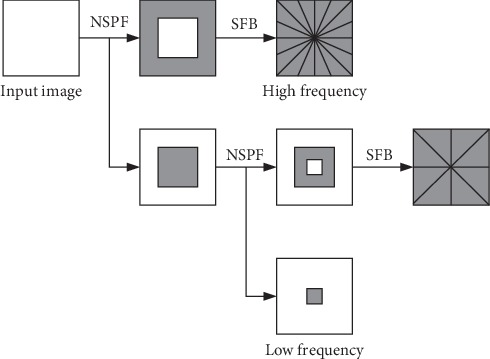
Schematic diagram of NSST decomposition.

The NSST decomposes the source image through the non-subsampled pyramid filter (NSPF) and the shift-invariant shear filter bank (SFB). NSPF can guarantee the shift-invariance and suppress the pseudo-Gibbs phenomenon, and SFB can achieve the directional localization.  Figure 1 is a schematic diagram of the NSST decomposition. NSST is recognized as a very reliable image fusion method with good local time-domain features, multidirectionality, and translation invariance. It can effectively extract the edge and detail information in the source image [2, 8]. On account of this, NSST was selected as the MST method of image fusion.

#### 2.1.2. Parameter-Adaptive Pulse-Coupled Neural Network (PAPCNN) and Improvement of the Parameter *β*

The key challenge in the traditional PCNN model is how to set free parameters such as connection strength, various amplitudes, and attenuation coefficients. To avoid difficulties in manually setting free parameters, in this paper, a parameter-adaptive PCNN (PAPCNN) model [7] was proposed to fuse the high-frequency coefficients obtained by NSST decomposition.

The PAPCNN model is described as follows:

(1) $$\begin{array}{c} F_{ij}\left[n\right]=S_{ij}, \end{array}$$

(2) $$\begin{array}{c} L_{ij}\left[n\right]=V_{L}\underset{kl}{\sum }W_{ijkl}Y_{kl}\left[n-1\right], \end{array}$$

(3) $$\begin{array}{c} U_{ij}\left[n\right]=e^{-\alpha_{f}}U_{ij}\left[n-1\right]+F_{ij}\left[n\right]\left(1+\beta L_{ij}\left[n\right]\right), \end{array}$$

(4) $$\begin{array}{c} Y_{ij}\left[n\right]=\left\{\begin{array}{cc} 1, & \text{if }U_{ij}\left[n\right]>E_{ij}\left[n-1\right],\\ 0, & \text{otherwise,} \end{array}\right. \end{array}$$

(5) $$\begin{array}{c} E_{ij}\left[n\right]=e^{-\alpha_{e}}E_{ij}\left[n-1\right]+V_{E}Y_{ij}\left[n\right]. \end{array}$$

**Figure 2:**
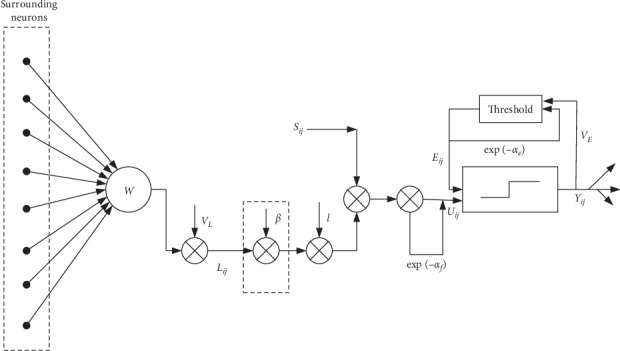
Structure of the PAPCNN model.

In the PAPCNN model mentioned above, *F*_*ij*_[*n*] and *L*_*ij*_[*n*] represent the input and connection input of the neurons at the position of the iteration *n*, respectively.  Figure 2 shows the structure of the PAPCNN model.

There are five parameters in the PAPCNN model: the attenuation coefficient *α*_*f*_ of the dynamic threshold *E*, the connection strength *β*, the amplitude *V*_*L*_ of the connected input, the attenuation coefficient *α*_*e*_ of the internal activity *U*, and the amplitude *V*_*E*_ of the dynamic threshold *E*. Also, it can be observed from (1) to (5) that *β* or *V*_*L*_ only serves as the weight of ∑_*kl*_*W*_*ijkl*_*Y*_*kl*_[*n* − 1], so they can be treated as a whole *βV*_*L*_ in the PAPCNN model. Supposing that *λ*=*βV*_*L*_ represents the weighted connection strength, we analyze the value of parameter *V*_*L*_ according to the literature [6] and assume *V*_*L*_=1 without influence on the final experimental results; therefore, there are four parameters: *α*_*f*_, *α*_*e*_, *V*_*E*_, and *λ*.

In this paper, we have adjusted the parameter *β*, i.e., the connection strength between neurons. Because the value of *V*_*L*_ is fixed, the larger the value of *β* is, the greater the neuron is affected by its neighboring neurons, and the more intense the fluctuation of its internal activity *U*_*ij*_[*n*]. Generally, the larger value of *β* tends to cause low-light neurons to ignite; conversely, the smaller value of *β* may reduce the ability to capture the neighboring neurons. To coordinate the synchronous ignition characteristics of the PAPCNN model, an optimization method is introduced in this paper to search the value of *β* [9]:

(6) $$\begin{array}{c} \begin{array}{cc} \underset{\beta }{\min} & \overset{2}{\underset{c=1}{\sum }}\lambda_{c}{\underset{x\in X_{c}}{\sum }\left(I_{x}-m_{c}\left(n\right)\right)}^{2},\\ s.t. & {\text{X}}_{1}=\left\{\left.y\right|U_{y}\left(n\right)\leq E_{y}\left(n-1\right)\right\}\cap X,\\ & {\text{X}}_{2}=\frac{X}{X_{1}},0\leq \beta \leq 1, \end{array} \end{array}$$

where *λ*_1_ and *λ*_2_ are the weight coefficients, set to 1 and *X* indicates the set of neighboring neurons and is generally calculated by

(7) $$\begin{array}{c} X=\left\{\left.ij\right|L_{ij}\left(n\right)>0\right\}\cap \left\{\left.ij\right|Y_{ij}\left(n\right)=0\right\}, \end{array}$$

where *m*_1_(*n*) and *m*_2_(*n*) indicate, respectively, the mean value corresponding to the unfired and ignition areas, as shown in the following equation:

(8) $$\begin{array}{c} m_{c}\left(n\right)=\frac{\sum_{ij\in \Omega_{c}}F_{ij}}{\sum_{ij\in \Omega_{c}}1},\;c=1,2, \end{array}$$

where *Ω*_1_={*ij*|*Y*_*ij*_(*n* − 1)=0} and *Ω*_2_={*ij*|*Y*_*ij*_(*n* − 1)=0}. It can be seen from equation (6) that *β*, as an implicit parameter, changes the optimal value of the objective function. It essentially regulates the internal ignition activity *U* of the neighboring neurons, and later, by comparison with the threshold *E*, the neighboring neurons divided into two categories: *X*_1_ and *X*_2_. To this end, its corresponding gray value information and the dispersion degree of mean value in equation (8) were considered to determine the optimal connection coefficient *β*. To facilitate the calculation, the search method of increasing the step size Δ*β* was adopted.

#### 2.1.3. Convolutional Sparse Representation (CSR)

Convolutional sparse representation is a convolutional form of sparse representation, that is, the convolutional sum of the filter dictionary and the characteristic response is used instead of the product of the redundancy dictionary and the sparse coefficient, so that the image is sparsely coded in the unit of “entirety.” The convolutional sparse representation model can be expressed as

(9) $$\begin{array}{c} \arg\min_{x_{m}}\frac{1}{2}{\left\|\sum_{m}d_{m}⊗x_{m}-s\right\|}_{2}^{2}+\lambda {\sum_{m}\left\|x_{m}\right\|}_{1}, \end{array}$$

where {*d*_*m*_} represents the M-dimensional convolution dictionary; ⊗ represents the symbol of the convolution operation; {*x*_*m*_} represents the characteristic response; *s* represents the source image; the alternating direction method of multipliers (ADMMs) is a dual convex optimization algorithm, which can solve the convex programming problem with separable structure by solving alternately several subproblems. In [10], considering that the ADMM algorithm could desirably solve the problem of Basis Pursuit De-Noising (BPDN), a Fourier domain ADMM algorithm was proposed to solve the solving problem of the convolutional sparse model. Among them, dictionary learning is defined as the optimization problem of

(10) $$\begin{array}{c} \begin{array}{cc} \underset{\left\{d_{m}\right\},\left\{x_{m}\right\}}{\arg\min} & \frac{1}{2}{\left\|\overset{M}{\underset{m-1}{\sum }}d_{m}⊗x_{m}-s\right\|}_{2}^{2}+\lambda {\overset{M}{\underset{m-1}{\sum }}\left\|x_{m}\right\|}_{1},\\ s.t. & {\left\|d_{m}\right\|}_{2}=1. \end{array} \end{array}$$

The first application of the convolutional sparse representation to image fusion is described in the literature [5], which regards CSR as an alternative form of SR, to achieve sparse representation of the entire image, rather than partial image patches. The convolutional sparse representation algorithm overcomes the shortcomings of traditional sparse representation with limited ability to preserve details and high sensitivity to registration errors. We believe that it is also effective for the fusion of low-frequency coefficients. In particular, the application of the CSR model is very effective for the fusion of the low-frequency coefficients obtained by MST. The low-frequency coefficients obtained after the NSST decomposition represent the approximate description of the image, and there is a large number with the approximation of 0, which can sparsely represent the low-frequency information of the image. Based on the above considerations, the CSR model was introduced into the fusion of MST low-frequency coefficients.

### 2.2. Implementation of NSST-PAPCNN-CSR

**Figure 3:**
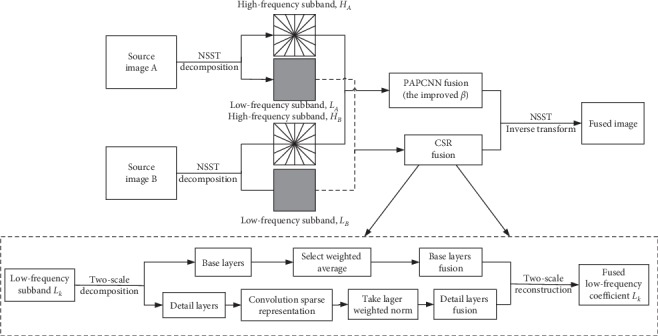
NSST-PAPCNN-CSR algorithm flow chart.

Figure 3 shows the specific steps of image fusion. The preregistered multimodal source images were fused, and the detailed fusion method includes four steps: NSST decomposition, fusion of high-frequency coefficients, fusion of low-frequency coefficients, and NSST reconstruction.

 
*Step 1.* NSST decomposition.
  The L-level NSST was used to decompose the source images A and B to obtain their coefficients {*H*_*A*_^*l*,*k*^, *L*_*A*_} and {*H*_*B*_^*l*,*k*^, *L*_*B*_}, respectively, where *H*_*A*_^*l*,*k*^ is a high-frequency coefficient of image A in the decomposition order *l* and the decomposition direction *k* and *L*_*A*_ is the low-frequency coefficient of image A. For image B, *H*_*B*_^*l*,*k*^ and *L*_*B*_ had the same meaning.
 
*Step 2.* Fusion of high-frequency coefficients.
  The PAPCNN model proposed in Section 2.1.2 was applied to the fusion of high-frequency coefficients [11]. Based on the discussion in Section 2.1.2, the absolute value graph of high-frequency coefficients was taken as the network input, namely, the feed input was *F*_*ij*_[*n*]=|*H*_*s*_^*l*,*k*^|,  *S* ∈ {*A*, *B*}. The activity level of high-frequency coefficients was measured by the total emission time throughout the iteration. According to the PAPCNN model described by Formulas (1)–(5), the trigger time was accumulated by adding the following steps at the end of each iteration:

(11) $$\begin{array}{c} T_{ij}\left[n\right]=T_{ij}\left[n-1\right]+Y_{ij}\left[n\right]. \end{array}$$

  The excitation time of each neuron was *T*_*ij*_[*n*] and *N* is the total number of iterations, corresponding to high-frequency coefficients *H*_*A*_^*l*,*k*^ and *H*_*B*_^*l*,*k*^. Their PAPCNN time could be calculated and expressed as *T*_*A*,*ij*_^*l*,*k*^[*n*] and *T*_*B*,*ij*_^*l*,*k*^[*n*]. The fused coefficient was obtained in the following way:

(12) $$\begin{array}{c} H_{F}^{l,k}\left(i,j\right)=\left\{\begin{array}{cc} H_{A}^{l,k}\left(i,j\right), & \text{if }{\text{T}}_{A,ij}^{l,k}\left[N\right]\geq T_{B,ij}^{l,k}\left[N\right],\\ H_{B}^{l,k}\left(i,j\right), & \text{otherwise}. \end{array}\right. \end{array}$$

  The above formula shows that the coefficient with the larger number of ignitions was the final high-frequency fusion coefficient. The optimal value of the object function was acquired by adjusting the size of the implicit parameter *β*, to obtain the optimal high-frequency fusion coefficient.
 
*Step 3.* Fusion of low-frequency coefficients.
  The fusion strategy of low-frequency coefficients also has a great influence on the final fusion quality. The convolutional sparse representation method was used to fuse low-frequency coefficients [12]. Suppose there were low-frequency coefficients after the decomposition of *k* source images and they were set *L*_*k*_, *k* ∈ {1, ..., *K*} and suppose a set of dictionary filters *d*_*m*_, *m* ∈ {1, ..., *M*}. The specific implementation steps of the low-frequency coefficients fusion based on CSR are shown in Figure 3.
 
*Step 4.* NSST reconstruction.

Finally, the inverse NSST reconstruction was performed on the fusion band {*H*_*F*_^*l*,*k*^, *L*_*F*_} to obtain the fused image *F*.

## 3. Experiments and Analysis

### 3.1. Experimental Settings

#### 3.1.1. Source Images

To verify the effectiveness of the proposed algorithm, 70 pairs of source pictures were used in the experiment. All of these source images are collected from the database of the Whole Brain Atlas of Harvard Medical School [13] and the Cancer Imaging Archive (TCIA) [14]. 50 pairs of source images from the database of Whole Brain Atlas include 10 pairs of CT and MR images, 10 pairs of T1-weighted (MR-T1) and T2-weighted (MR-T2) images, 15 pairs of MR and PET images, and 15 pairs of MR and SPECT images. 20 pairs of source images from the database of TCIA include 10 pairs of CT and MR images and 10 pairs of T1-weighted (MR-T1) and T2-weighted (MR-T2) images. All the source images have the same spatial resolution of 256 × 256 pixels. The source images in each pair have been accurately registered.

#### 3.1.2. Objective Evaluation Metrics

The evaluation of image fusion quality is divided into subjective visual evaluation and objective index evaluation. The objective evaluation metrics is to select relevant indices to measure the effect of human visual system on image quality perception. To quantitatively evaluate the performance of different methods, six accepted objective fusion evaluation indices were selected in the experiment, i.e., entropy (EN) [15], edge information retention (*Q*^AB/*F*^) [16], mutual information (MI), average gradient (AG), space frequency (SF), and standard deviation (SD) [17]. Entropy characterizes the amount of information available in the source image and the fused image; edge information retention characterizes the transfer amount of edge detail information in the source images injected into the fused image; mutual information is used to measure the information of the fused image contained in the used image; average gradient can be used to represent the sharpness of the image, and the larger the value, the clearer the image; space frequency reflects the overall activity of the image in the space domain, and its size is proportional to the image fusion effect; standard deviation reflects the dispersion degree of the pixel value and mean value of the image, and the greater the deviation, the better the quality of the image. In general, for all the above six metrics, a larger score indicates a better performance.

#### 3.1.3. Methods for Comparison

The proposed fusion method was compared with the existing five representative methods: the multimodal image fusion based on parameter‐adaptive pulse‐coupled neural network (NSST-PAPCNN) [7], the multimodal image fusion based on convolutional sparse representation (CSR) [5], the multimodal image fusion based on multiscale transform and sparse representation (MST-SR) [18], the multimodal image fusion based on sparse representation and pulse‐coupled neural network (SR-PCNN) [19], and the multimodal image fusion based on non-subsampled contourlet transform and sparse representation and pulse‐coupled neural network (NSCT-SR-PCNN) [10].

#### 3.1.4. Clinical Significance

The four types of medical image fusion have different clinical application value. For example, the fusion of CT and MR images can clearly display the location image of lesions and significantly reduce the surgical risk of visualized craniocerebral operation and the side effect of radiotherapy for craniocerebral lesions; the fusion of MR and SPECT images can determine epilepsy lesions in the neocortex of the brain based on local cerebral blood flow changes. Therefore, medical image fusion can combine the advantages of various imaging techniques and is of great significance in the diagnosis and treatment of diseases.

### 3.2. Comparison with Other Image Fusion Methods

In this section, the proposed method (NSST-PAPCNN-CSR) is compared with other approaches on visual quality and objective assessment.

#### 3.2.1. Source Images from the Whole Brain Atlas of Harvard Medical School

The whole brain Atlas of Harvard Medical School is created by Keith and Johnson from Harvard Medical School. It includes brain samples of normal brain, cerebrovascular disease, brain tumor, degenerative disease, and other brain diseases. The same slice of the same brain is equipped with the registered CT, MR or MR-T1, MR-T2 or PET, and SPECT images. Each pair of source images used in this section are obtained by different imaging methods for the same slice (slice thickness is generally 3 mm or 5 mm) in the same brain at the same angle.

**Figure 4:**
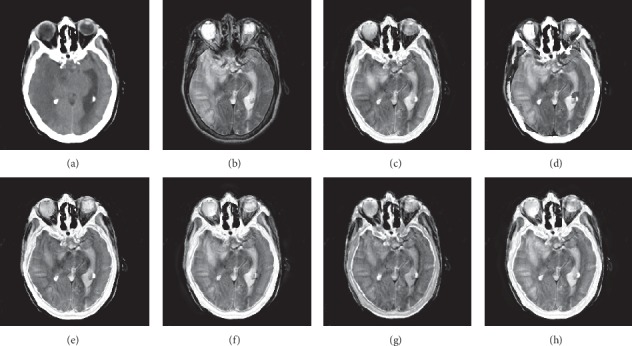
CT and MR medical image fusion results. (a) CT original image. (b) MR original image. (c) NSST-PAPCNN. (d) CSR. (e) MST-SR. (f) NSCT-SR-PCNN. (g) SR-PCNN. (h) Proposed.

**Figure 5:**
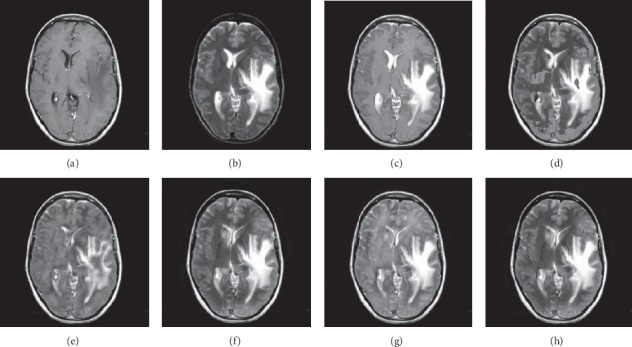
CT and MR medical image fusion results. (a) CT original image. (b) MR original image. (c) NSST-PAPCNN. (d) CSR. (e) MST-SR. (f) NSCT-SR-PCNN. (g) SR-PCNN. (h) Proposed.

**Figure 6:**
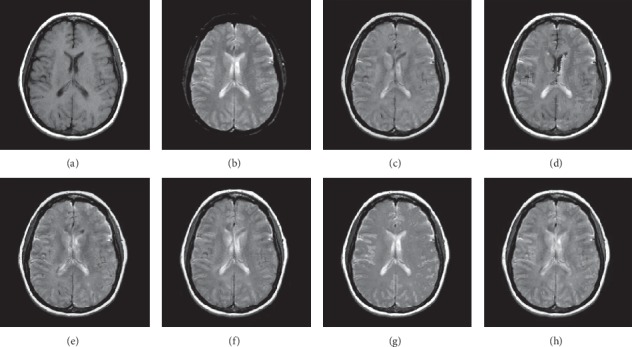
MR-T1 and MR-T2 medical image fusion results. (a) MR-T1 original image. (b) MR-T2 original image. (c) NSST-PAPCNN. (d) CSR. (e) MST-SR. (f) NSCT-SR-PCNN. (g) SR-PCNN, (h) Proposed.

**Figure 7:**
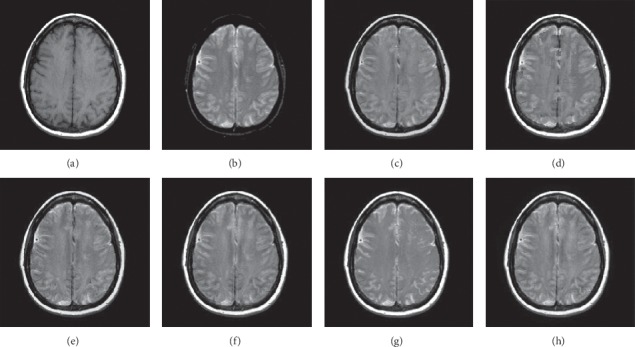
MR-T1 and MR-T2 medical image fusion results. (a) MR-T1 original image. (b) MR-T2 original image. (c) NSST-PAPCNN. (d) CSR. (e) MST-SR. (f) NSCT-SR-PCNN. (g) SR-PCNN, (h) Proposed.

**Figure 8:**
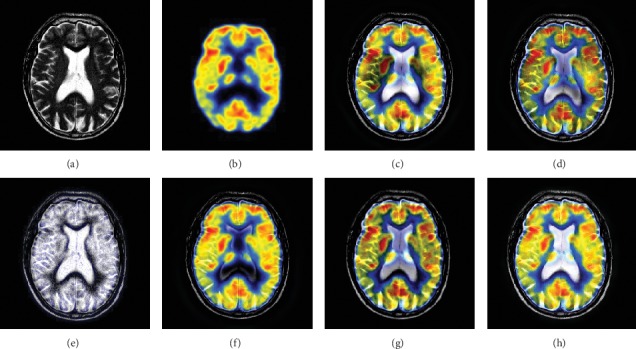
MR and PET medical image fusion results. (a) MR original image. (b) PET original image. (c) NSST-PAPCNN. (d) CSR. (e) MST-SR. (f) NSCT-SR-PCNN. (g) SR-PCNN. (h) Proposed.

**Figure 9:**
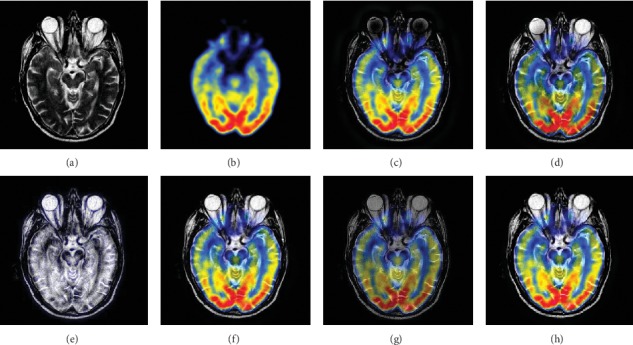
MR and PET medical image fusion results. (a) MR original image. (b) PET original image. (c) NSST-PAPCNN. (d) CSR. (e) MST-SR. (f) NSCT-SR-PCNN. (g) SR-PCNN. (h) Proposed.

**Figure 10:**
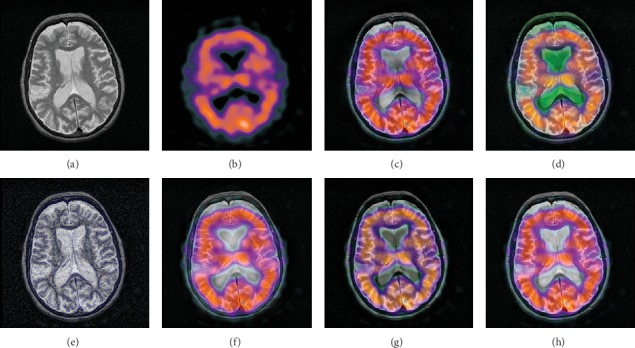
MR and SPECT medical image fusion results. (a) MR original image. (b) SPECT original image. (c) NSST-PAPCNN. (d) CSR. (e) MST-SR. (f) NSCT-SR-PCNN. (g) SR-PCNN. (h) Proposed.

**Figure 11:**
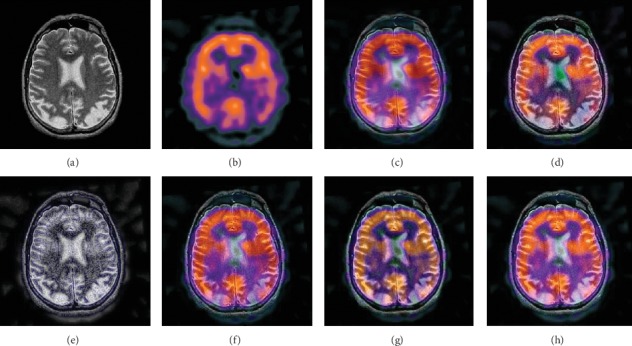
MR and SPECT medical image fusion results. (a) MR original image. (b) SPECT original image. (c) NSST-PAPCNN. (d) CSR. (e) MST-SR. (f) NSCT-SR-PCNN. (g) SR-PCNN. (h) Proposed.

In this experiment, 50 pairs of brain source images in different states were selected for fusion, including 10 pairs of CT/MR images, 10 pairs of MR-T1/MR-T2 images, 15 pairs of MR/PET images, and 15 pairs of MR-SPECT images. We show the fusion results of some of the source images. The fused images are shown in Figures 4–11, and their objective quality evaluation indicators are listed in Table 1.

When the source images come from the whole brain Atlas of Harvard Medical School, the proposed method performs well better than other five contrast methods on both energy preservation, detail extraction, and color preservation, as shown in Figures 4–11. Table 1 lists the objective assessment of different fusion methods on four categories of medical image fusion problems. The average score of each method over all the testing images in each fusion problem is reported. For each index, its maximum value is denoted in bold and italics, and the second biggest value is underlined. In this paper, the RMSE (root-mean-square error) of each index mean of the algorithm is calculated to verify the validity of the data of each index mean of the proposed algorithm. It can be seen from Table 1 that when the source image comes from the whole brain Atlas of Harvard Medical School, the RMSE of each index of the proposed algorithm does not fluctuate more than 1, which has strong data validity. It is known from the objective indices listed in Table 1 that the proposed algorithm had better performance in the MI and SD indices than the other five contrast algorithms. Among them, MI was 8.6% higher and SD 17.5% higher than the average of the five contrast algorithms. NSST-PAPCNN-CSR is not always the best one among the five contrast algorithms in each individual evaluation indicator, but it never ranked less than the top two.

Overall, for the various source images from the whole brain Atlas of Harvard Medical School, the NSST-PAPCNN-CSR algorithm not only achieved better fusion performance visually in edge sharpness, change intensity, and contrast but also performed excellently in objective fusion indicators.

#### 3.2.2. Source Images from the Cancer Imaging Archive (TCIA)

The Cancer Imaging Archive (TCIA) is an open-access database of medical images for cancer research. It is usually composed of common diseases (such as lung cancer and brain cancer). The image morphology includes CT, MR, and so on. It also provides image related supporting data, such as the number and date of brain slices. Each pair of source images used in this section are obtained by different imaging methods for the same slice in the same brain at the same angle.

**Figure 12:**
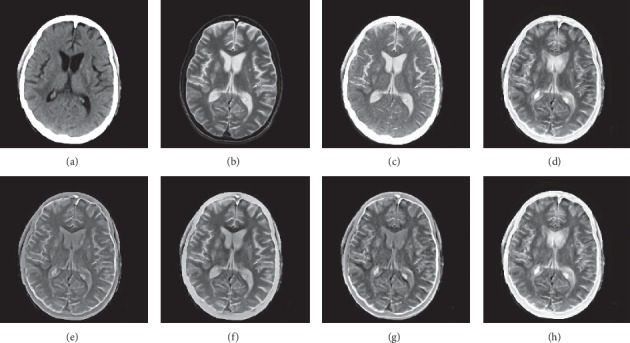
CT and MR medical image fusion results. (a) CT original image. (b) MR original image. (c) NSST-PAPCNN. (d) CSR. (e) MST-SR. (f) NSCT-SR-PCNN. (g) SR-PCNN. (h) Proposed.

**Figure 13:**
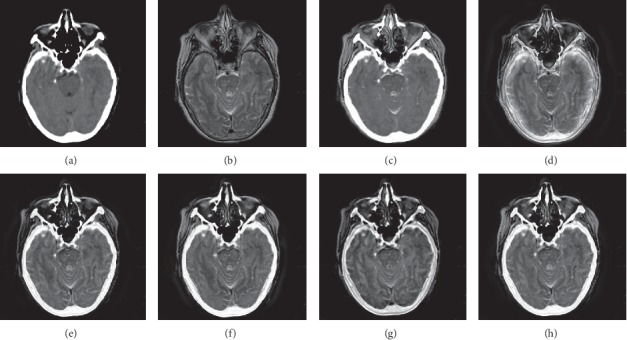
CT and MR medical image fusion results. (a) CT original image. (b) MR original image. (c) NSST-PAPCNN. (d) CSR. (e) MST-SR. (f) NSCT-SR-PCNN. (g) SR-PCNN. (h) Proposed.

**Figure 14:**
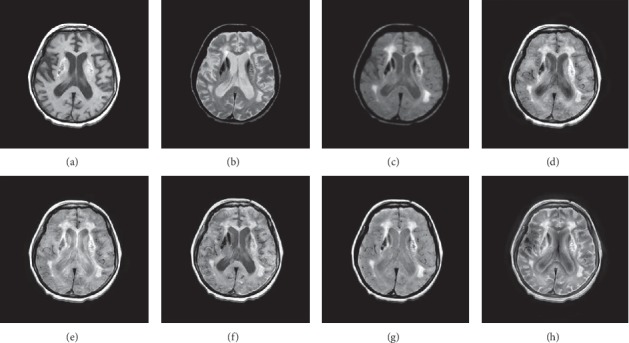
MR-T1 and MR-T2 medical image fusion results. (a) MR-T1 original image. (b) MR-T2 original image. (c) NSST-PAPCNN. (d) CSR. (e) MST-SR. (f) NSCT-SR-PCNN. (g) SR-PCNN. (h) Proposed.

**Figure 15:**
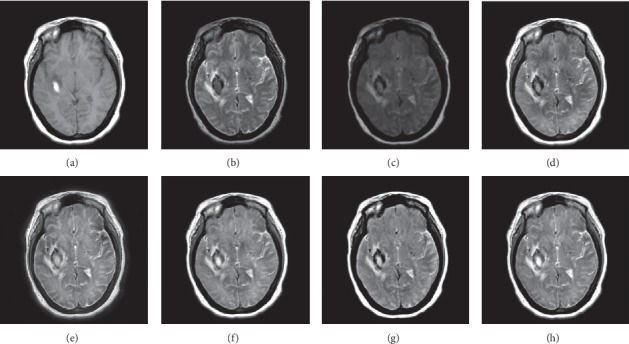
MR-T1 and MR-T2 medical image fusion results. (a) MR-T1 original image. (b) MR-T2 original image. (c) NSST-PAPCNN. (d) CSR. (e) MST-SR. (f) NSCT-SR-PCNN. (g) SR-PCNN. (h) Proposed.

In this experiment, because TCIA has few suitable PET and SPECT images to do fusion experiments, 10 pairs of CT/MR and 10 pairs of MR-T1/MR-T2 brain source images in different states were selected for fusion. We show the fusion results of some of the source images. The fused images are shown in Figures 12–15, and their objective quality evaluation indicators are listed in Table 1.

When the source images come from the Cancer Imaging Archive (TCIA), the proposed method performs well better than other five contrast methods on both energy preservation and detail extraction and color preservation, as shown in Figures 12–15. The objective assessments of different fusion methods on two categories of medical image fusion problems are listed in Table 1. The average score of each method over all the testing images in each fusion problem is reported. In this paper, the RMSE (root-mean-square error) of each index mean of the algorithm is calculated to verify the validity of the data of each index mean of the proposed algorithm. It can be seen from Table 1 that when the source image comes from the Cancer Imaging Archive (TCIA), the RMSE of each index of the proposed algorithm does not fluctuate more than 1, which has strong data validity. It is known from the objective indices listed in Table 1 that the proposed algorithm had better performance in the *Q*^AB/*F*^ and AG and SD indices than the other five contrast algorithms. Among them, *Q*^AB/*F*^ was 17.9% higher, AG 8.8% higher, and SD 7.7% higher than the average of the five contrast algorithms. NSST-PAPCNN-CSR is not always the best one among the five contrast algorithms in each individual evaluation indicator, but it never ranked less than the top two.

In summary, for the various source images from the Whole Brain Atlas of Harvard Medical School and the Cancer Imaging Archive (TCIA), the NSST-PAPCNN-CSR algorithm not only achieved good fusion effect visually in terms of edge sharpness, change intensity, and contrast but also performed excellently in objective fusion indicators.

## 4. Conclusion

A novel NSST domain medical image fusion method was proposed and there were mainly two innovations. First, a PAPCNN model was introduced into the fusion of high-frequency coefficients. All free parameters in the model were calculated adaptively according to the input high-frequency coefficients; furthermore, the parameter *β* was adjusted to its optimal value and the synchronous ignition characteristics of the PAPCNN model were coordinated even better. Second, convolutional sparse representation was applied to low-frequency coefficient fusion. It solved two problems existing in sparse representation, namely, limited ability of detail preservation and high sensitivity to mismatch. Thus, it was able to fuse low-frequency coefficients better. 70 pairs of multimodal source images and five kinds of contrast algorithms were used to conduct experiments. The results show that the proposed method has excellent performance in terms of visual perception and objective effect evaluation. The NSST-PAPCNN-CSR algorithm still has potential applications in multifocus image fusion, infrared/visible image fusion, and other image fusion problems.

## Figures and Tables

**Table 1:** Objective assessment of different methods on medical image fusion.

The database	Images	Metrics	NSST-PAPCNN	CSR	MST-SR	NSCT-SR-PCNN	SR-PCNN	Proposed	RMSE (proposed)
The Whole Brain Atlas Of Harvard Medical School	CT/MR	EN	3.1249 (1)	2.9919	2.8641	3.0749	2.7759	3.0767 (2)	0.0262
		*Q* ^AB/*F*^	0.4587	0.4427	0.4325	0.4801 (2)	0.4408	**0.4839 (1)**	0.0304
		MI	0.8093	0.8079	0.7779	0.8333 (2)	0.7396	**0.8599 (1)**	0.0156
		SF	27.8907	28.4779 (2)	27.6372	27.9712	27.4416	**29.3626 (1)**	0.6256
		AG	6.9390	7.1364 (2)	7.1117	6.9873	6.5669	**7.1671 (1)**	0.0392
		SD	110.4631	110.6766 (2)	109.7036	109.4976	108.0036	**111.0203 (1)**	0.3939
	MR-T1/MR-T2	EN	3.0751	3.0954	3.1193	3.1564 (2)	2.9382	3.2436 (1)	0.0593
		*Q* ^AB/*F*^	0.4223	0.4428	**0.5345 (1)**	0.4327	0.4241	0.4926 (2)	0.0587
		MI	1.0879	1.0932	1.1119	1.1247 (2)	1.0830	**1.1675 (1)**	0.1132
		SF	26.8878	**27.8143 (1)**	27.4827	27.3243	22.6816	27.6630 (2)	0.7533
		AG	4.4525	4.9000 (2)	4.8198	4.7360	3.1408	**4.9541 (1)**	0.3882
		SD	109.2559	109.3714	109.2907	109.0063	110.1740 (2)	**111.4005 (1)**	0.2773
	MR/PET	EN	3.2575	3.1860	3.2732 (2)	3.1620	3.1728	3.3962 (1)	0.3524
		*Q* ^AB/*F*^	0.5213	0.5783	0.6148 (2)	0.5216	0.6125	0.6621 (1)	0.2436
		MI	1.6716 (2)	1.6604	1.6708	1.5385	1.6688	1.7078 (1)	0.3849
		SF	27.0508	29.2171	**31.8184 (1)**	28.1990	29.5717	30.0488 (2)	0.0968
		AG	5.1704	6.3783	6.8126 (2)	5.8611	6.5209	**7.8436 (1)**	0.7054
		SD	112.5116	112.5743 (2)	111.8941	110.2727	111.9179	**114.7089 (1)**	0.8578
	MR/SPECT	EN	3.5580	3.5357	3.8857 (1)	3.6305	3.4818	3.6897 (2)	0.5361
		*Q* ^AB/*F*^	0.4561	0.5465	0.7211 (2)	0.5564	0.5521	**0.7426 (1)**	0.0389
		MI	1.3484	1.3491	1.3511	1.3663 (2)	1.3658	**1.4261 (1)**	0.4157
		SF	23.9815	25.2731	**27.7746 (1)**	25.1314	24.6361	25.4938 (2)	0.3981
		AG	4.1747	4.7093	4.7542	4.8058 (2)	4.5293	**4.9342 (1)**	0.6432
		SD	**109.8246 (1)**	108.6124	109.4126	109.3390	108.6478	109.5151 (2)	0.8745

The Cancer Imaging Archive	CT/MR	EN	2.3873	2.5588 (1)	2.4780	2.5250	2.4138	2.5412 (2)	0.0348
		*Q* ^AB/*F*^	0.2823	0.3242	0.3400 (2)	0.3022	0.3083	**0.3461 (1)**	0.0452
		MI	0.4202	0.4998	0.5359 (2)	0.5328	0.5302	**0.5680 (1)**	0.0799
		SF	23.5399	**27.1924 (1)**	25.3864	24.4104	23.8955	25.5036 (2)	0.5346
		AG	5.5653	5.0692	5.5261	5.6596 (2)	5.5149	**5.6693 (1)**	0.0569
		SD	101.6034	111.2382 (2)	103.4071	102.6671	103.8596	**112.3046 (1)**	0.4832
	MR-T1/MR-T2	EN	3.0059	3.0495	3.0649 (2)	2.9463	2.9340	3.1736 (1)	0.6203
		*Q* ^AB/*F*^	0.3183	0.4462	0.4928 (2)	0.4385	0.4061	**0.5103 (1)**	0.1108
		MI	0.8798	1.0100	**1.1441 (1)**	1.0069	0.9576	1.1064 (2)	0.7673
		SF	22.3876	26.9720 (2)	26.0597	26.9641	26.1218	**27.4718 (1)**	0.8564
		AG	3.4087	5.0089 (2)	4.9964	5.0070	5.0174	**5.1650 (1)**	0.1694
		SD	106.2062	108.2755	107.4503	109.2019 (2)	108.0475	**109.3821 (1)**	0.8521

## Data Availability

The data used to support the findings of this study are included within the article.
